# Preface

**DOI:** 10.2188/jea.17.S1

**Published:** 2008-01-30

**Authors:** Masaki Nagai, Shuji Hashimoto, Kiyosu Taniguchi

**Affiliations:** 1 Department of Public Health, Saitama Medical University Faculty of Medicine.; 2 Department of Hygiene, Fujita Health University School of Medicine.; 3 Infectious Disease Surveillance Center, National Institute of Infectious Diseases.

Before the Law Concerning the Prevention of Infectious Diseases and Medical Care for Patients of Infections (hereafter referred to as the Infectious Diseases Control Law) was enacted in 1999, a research group was organized to make proposals regarding a surveillance system in compliance with the new law. The authors, as the members of the group, had made several recommendations for the surveillance system. The first recommendation was how many sentinel clinics or hospitals were necessary, and how they were to be selected and assigned from all the clinics and hospitals in Japan. As the recommended system was expected to be able to make an acceptable estimation of the total number of patients nationwide based on the number of patients reported from the sentinels, the research group proposed a statistical method to perform the estimation. These accomplishments were published in a report in Japanese (Table 1. No. 1). The second recommendation was for establishing the alert system for the surveillance. The meaning and definition of alert and early warning were established, and the effective critical values for getting on/off the alert/early warning for each infection were suggested. These achievements were also published in another report in Japanese (Table 1. No. 2).

**Table 1.** Reports published by the research group for the evaluation of The National Epidemiological Surveillance of Infectious Diseases.

Authors		Title	Number of pages	Published year
1	Nagai M, Hashimoto S, Taniguchi K, Murakami Y	Report from the research group on the assignment of sentinels in The National Epidemiological Surveillance of Infectious Diseases	139	1999
2	Nagai M, Hashimoto S, Taniguchi K, Murakami Y, Tanihara S, Matumoto T, Yokota S, Kashiwagi S, Jou K, Aoki K, Fuchigami H	Report from the research group for the development of alert reporting system in The National Epidemiological Surveillance of Infectious Diseases	242	1999
3	Nagai M, Hashimoto S, Murakami Y, Osaka K, Shindo N, Shingai T, Fuchigami H	Alert reporting system and estimation of number of patients in whole nation	146	2001
4	Nagai M, Hashimoto S, Murakami Y, Osaka K, Shindo N, Fuchigami H	Alert reporting system and estimation of number of patients in whole nation, report 2	195	2002
5	Nagai M, Hashimoto S, Murakami Y, Taniguchi K, Osaka K, Fuchigami H	Alert reporting system and estimation of number of patients in whole nation, report 3	217	2003
6	Nagai M, Hashimoto S, Murakami Y, Taniguchi K, Osaka K, Shigematsu M, Kawado M	Alert reporting system and estimation of number of patients in whole nation, report 4	176	2004
7	Nagai M, Hashimoto S, Murakami Y, Taniguchi K, Shigematsu M, Kimura M, Tada Y, Kawado M, Izumida M	Alert reporting system and estimation of number of patients in whole nation, report 5	184	2005
8	Nagai M, Hashimoto S, Murakami Y, Kawado M, Taniguchi K, Shigematsu M, Kimura M, Tada Y, Yasui Y, Izumida M	Alert reporting system and estimation of number of patients in whole nation, report 6	152	2006
9	Nagai M, Hashimoto S, Murakami Y, Kawado M, Taniguchi K, Shigematsu M, Tada Y, Yasui Y, Ohta A, Izumida M	Alert reporting system and estimation of number of patients in whole nation, report 7	154	2007

The research group name is as indicated in the Report title for Nos. 1 and 2, and in the Table title for Nos. 3-9.

All the reports are written in Japanese.

Since the National Epidemiological Surveillance of Infectious Diseases (NESID), one of the main objectives of the new Infectious Diseases Control Law, was implemented in 1999, the research group has been continuously examining and evaluating the efficiency, efficacy and accuracy of the system from the main viewpoints of estimating the total number of patients across Japan and obtaining effective alerts for the target diseases at sentinel clinics and hospitals. The group has also been making recommendations for necessary amendments to the system for these purposes in each report published every year (Table 1. Nos. 3-9) and in several original reports. ^1^^-^^11^ In addition to the principal targets of the study, namely the total number of patients and effective alerts, the group has studied the effective utilization of the information on target diseases which must be reported for all cases and on other target diseases which must be reported by a limited number of sentinel hospitals primarily targeting inpatients in the NESID.

Though the main purpose of the NESID was not changed, the Infectious Diseases Control Law was amended in 2003 and the information process system was improved in 2006. On this occasion, the database accumulated since 1999 can be compiled and utilized. In the first report of this supplement, we will overview the purpose, the history and the structure of the NESID. And the evidences obtained from the analysis of the database are to be introduced in the following articles in this supplement.
